# Supplementary material for: The interactome of intact mitochondria by cross-linking mass spectrometry provides evidence for coexisting respiratory supercomplexes
Source: Mol Cell Proteomics. 2017 Dec 8;17(2):216–32. doi: 10.1074/mcp.RA117.000470 (PMC5795388; doi:10.1074/mcp.RA117.000470)
Supplement: Supplemental Data [file supp_RA117.000470_133922_0_supp_23978_fzffwf.zip › spectra_annotation/mito_DR_spectra_annotation/302-2-9-1-6-1.pdf]

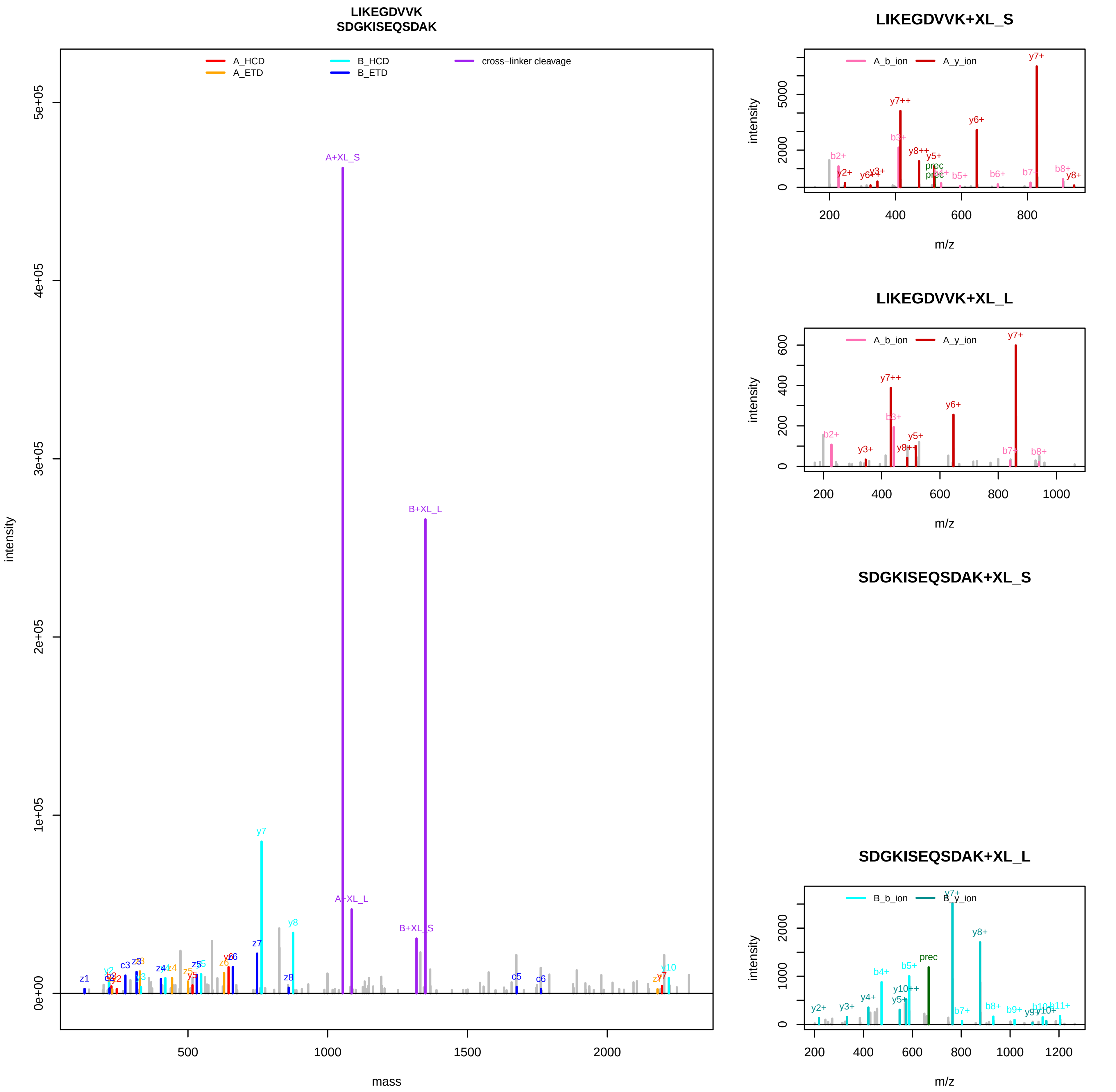

LIKEGDVVK
SDGKISEQSDAK
A_HCD
A_ETD
B_HCD
B_ETD
cross−linker cleavage
A+XL_S
B+XL_L
A+XL_L
B+XL_S
y7
y8
z7
z8
c3
z3
z4
z5
c5
c6
z1
y10
intensity
mass
0e+00
1e+05
2e+05
3e+05
4e+05
5e+05
500
1000
1500
2000
LIKEGDVVK+XL_S
A_b_ion
A_y_ion
y7+
y7++
y6+
y8++
y5+
prec
b2+
y2+
b3+
b5+
b6+
b7+
b8+
y8+
m/z
LIKEGDVVK+XL_L
SDGKISEQSDAK+XL_S
SDGKISEQSDAK+XL_L
B_b_ion
B_y_ion
y8+
b4+
b5+
y10++
y4+
y5+
y2+
y3+
b7+
b8+
b9+
